# Cerebrovascular thrombosis during pediatric ALL therapy: a case series highlighting temporal association with PEG-asparaginase exposure

**DOI:** 10.3389/fped.2026.1829820

**Published:** 2026-06-15

**Authors:** Chen Xu, Jianchun Lu, Weihao Ling, Bingbing Zhang, Huan Xu, Jihong Tang

**Affiliations:** 1 Department of Neurology, Children’s Hospital of Soochow University, Suzhou, Jiangsu, China; 2 Department of Neurology, Suzhou Wujiang District Children’s Hospital, Suzhou, Jiangsu, China

**Keywords:** acute lymphoblastic leukemia, antithrombin III, arterial ischemic stroke, cerebral venous sinus thrombosis, pediatric thrombosis, PEG-asparaginase

## Abstract

**Background:**

Cerebrovascular thrombosis is an uncommon but potentially life-threatening complication of chemotherapy in children with acute lymphoblastic leukemia (ALL). Although PEG-asparaginase–associated thrombosis has been widely recognized, the temporal pattern, laboratory evolution, and management characteristics of cerebrovascular events remain incompletely described.

**Methods:**

We conducted a retrospective single-center case series of pediatric patients with acute lymphoblastic leukemia (ALL) treated between January 2018 and December 2025. Clinical records, serial coagulation parameters, neuroimaging findings, treatment strategies, and outcomes of patients who experienced cerebrovascular thrombotic events during chemotherapy were reviewed descriptively.

**Results:**

Among 1,138 pediatric patients with ALL treated during the study period, six developed radiologically confirmed cerebrovascular thrombosis (incidence proportion, 0.53%; median age, 10 years). Five patients developed cerebral venous sinus thrombosis (CVST) and one developed acute ischemic stroke (AIS). Five of six events in this small cohort occurred within 5–17 days after PEG-asparaginase administration. At symptom onset, all patients exhibited markedly elevated D-dimer levels and reduced antithrombin III (AT-III) activity. Following treatment, D-dimer levels declined within 1–3 days, whereas AT-III activity recovered more gradually over 5–13 days. Four patients with CVST received therapeutic low-molecular-weight heparin and achieved favorable neurological outcomes. One patient with AIS underwent successful mechanical thrombectomy with substantial neurological improvement. No recurrent thrombotic events were observed during follow-up.

**Conclusions:**

In this case series, cerebrovascular thrombosis during pediatric ALL therapy showed a distinct temporal clustering pattern within 5–17 days after PEG-asparaginase exposure and was consistently associated with reduced AT-III activity and elevated D-dimer levels. These findings suggest a potential high-risk period during which focused coagulation monitoring may facilitate earlier diagnosis and timely therapeutic intervention.

## 1 Introduction

Acute lymphoblastic leukemia (ALL) is the most common malignancy in childhood, and advances in risk-adapted chemotherapy protocols have led to substantial improvements in survival rates over recent decades (1). As long-term outcomes continue to improve, treatment-related complications have become increasingly important determinants of morbidity and quality of life (2). Among treatment-related toxicities, cerebrovascular thrombosis has emerged as an important but potentially underrecognized complication during intensive chemotherapy for pediatric ALL (3).

Cerebrovascular thrombosis during pediatric ALL therapy, including cerebral venous sinus thrombosis (CVST) and acute arterial ischemic stroke (AIS), is uncommon but potentially devastating (4–6). These complications most frequently occur during intensive phases of chemotherapy, particularly induction and early consolidation, and often present with nonspecific neurological symptoms such as headache, seizures, altered consciousness, or focal neurological deficits (5, 7). Previous multicenter cohort studies have reported cerebrovascular thrombosis as an important complication in children undergoing ALL treatment; however, the reported incidence varies, and the underlying mechanisms remain incompletely understood (4, 5, 8, 9). Moreover, detailed descriptions of coagulation abnormalities, imaging characteristics, and interventional management strategies remain limited, particularly those integrating comprehensive laboratory and imaging correlation (10).

Several treatment-related and disease-related factors have been implicated in the pathogenesis of cerebrovascular events during ALL therapy. Pegylated asparaginase (PEG-asparaginase), a cornerstone of modern ALL regimens, is a well-recognized contributor to hypercoagulability through depletion of natural anticoagulants such as antithrombin III and fibrinogen (11, 12). Concomitant corticosteroid exposure, chemotherapy-induced endothelial injury, systemic inflammation, and the use of central venous catheters may further amplify thrombotic risk (10, 13). Despite growing recognition of these associations, optimal management strategies for cerebrovascular thrombosis in pediatric ALL remain poorly defined.

In this context, we conducted a retrospective case series at a tertiary pediatric center to characterize cerebrovascular thrombosis occurring during chemotherapy for pediatric ALL. By integrating detailed clinical presentation, laboratory coagulation profiles, neuroimaging findings, treatment approaches, and long-term neurological outcomes, we aimed to provide a focused analysis of chemotherapy-associated cerebrovascular events. In particular, this study highlights coagulation abnormalities at symptom onset and reports the feasibility of mechanical thrombectomy in a selected pediatric patient with acute large-vessel arterial occlusion, thereby contributing clinically relevant data to an area with limited existing evidence (14). Unlike prior studies primarily focused on incidence estimates or isolated thrombotic events, our study integrates temporal patterns after PEG-asparaginase exposure, serial coagulation kinetics, neuroimaging findings, and multidisciplinary management within a single pediatric ALL cohort.

We observed a clinically relevant high-risk interval following PEG-asparaginase exposure.

## 2 Materials and Methods

### 2.1 Study design and setting

This was a single-center retrospective case series conducted in the Department of Hematology, Children's Hospital of Soochow University, a tertiary pediatric referral center in China. We retrospectively reviewed the electronic medical record system for pediatric patients with acute lymphoblastic leukemia (ALL) treated at Children's Hospital of Soochow University between January 2018 and December 2025. Potential cases were screened through hospitalization records, neuroimaging reports, neurology consultation records, and discharge diagnostic coding. Patients with radiologically confirmed cerebrovascular thrombosis occurring during chemotherapy were included in the final analysis.

### 2.2 Patient selection

#### 2.2.1 Inclusion criteria

Patients were eligible for inclusion if they met all of the following criteria:

1. age younger than 18 years at the time of leukemia diagnosis;
2. a confirmed diagnosis of acute lymphoblastic leukemia;
3. development of acute neurological symptoms during chemotherapy;
4. radiologically confirmed cerebrovascular events on brain magnetic resonance imaging (MRI).

#### 2.2.2 Exclusion criteria

Patients were excluded if the cerebrovascular event:

1. occurred after hematopoietic stem cell transplantation;
2. was associated with known congenital cerebrovascular malformations; or
3. was attributable to traumatic brain injury or clearly identified infectious etiologies.

## 3 Data collection

Clinical data were retrospectively extracted from electronic medical records. Collected variables included demographic characteristics (age and sex), leukemia immunophenotype, treatment protocol, and the phase of chemotherapy at the time of neurological event.

Neurological manifestations at presentation—such as headache, seizures, altered consciousness, and focal neurological deficits—were systematically recorded, along with the timing of symptom onset relative to chemotherapy administration. Detailed information regarding chemotherapy exposure was collected, including the use of pegylated asparaginase and corticosteroids. For patients who received pegylated asparaginase, the interval between the last dose and the onset of cerebrovascular symptoms was specifically documented.

Comprehensive inherited thrombophilia screening was not routinely performed because of the retrospective design and variability in clinical practice during the study period.

## 4 Laboratory assessment

Laboratory parameters reflecting coagulation and inflammatory status were collected at symptom onset or from the closest available prior testing. These included plasma D-dimer (*μ*g/L, reference <500 μg/L), antithrombin III (AT-III) activity, platelet count, prothrombin time, and activated partial thromboplastin time.

For patients receiving anticoagulation, follow-up measurements of AT-III and D-dimer were collected to evaluate recovery trends.

AT-III recovery was defined as a sustained increase from the lowest documented value during hospitalization after treatment initiation, whereas normalization referred to return to the institutional reference range (80%–120%).

Parameters recorded included:

1. AT-III recovery post-anticoagulation (time to increase, normalization status, recovery relative to PEG-asparaginase administration);
2. D-dimer decline after initiation of therapy;
3. Use of AT-III replacement;
4. LMWH-only treatment recovery.

Patients with ALL underwent evaluation for CNS infection when clinically indicated; no evidence of CNS infection was found. Because of the retrospective nature of the study, follow-up measurements of AT-III activity and D-dimer were not performed at predefined protocolized intervals, but were obtained according to clinical condition, physician discretion, and treatment response during hospitalization and follow-up.

### 4.1 Neuroimaging evaluation

Patients with ALL underwent brain MRI for diagnosis. Imaging included DWI, ADC maps, and vascular imaging via MRA or MRV as clinically indicated. Cerebrovascular events were classified as CVST or acute arterial ischemic stroke (AIS) based on imaging. Studies were reviewed by experienced pediatric neuroradiologists.

### 4.2 Treatment and management

Routine pharmacologic thromboprophylaxis was not administered during chemotherapy at our institution during the study period. Treatment strategies were individualized and determined by a multidisciplinary team comprising pediatric hematologists, neurologists, neurointerventional specialists, intensivists, and radiologists. Therapeutic-dose anticoagulation therapy with low-molecular-weight heparin (LMWH) was initiated for selected patients with CVST unless contraindicated because of severe thrombocytopenia or declined by the patient's family.

At our institution, platelet counts were generally maintained above 30–50 × 10⁹/L during therapeutic anticoagulation, depending on bleeding risk and clinical status. For invasive neurointerventional procedures, platelet transfusion support was administered when necessary, and therapeutic LMWH was generally initiated only after exclusion of intracranial hemorrhage and recovery of platelet counts to clinically acceptable levels.

One patient with acute arterial ischemic stroke caused by large-vessel occlusion underwent emergency mechanical thrombectomy after multidisciplinary evaluation. Procedural details, peri-procedural management, angiographic findings, and clinical outcomes were recorded.

### 4.3 Outcome assessment and follow-up

Neurological outcomes during hospitalization and at follow-up were evaluated. Outcomes were assessed based on neurological recovery and functional status. Follow-up brain imaging was performed as needed to evaluate lesion evolution or recanalization.

The follow-up duration was recorded in months, and the survival status was documented for all patients.

## 5 Statistical analysis

Continuous variables are presented descriptively as medians and ranges, whereas categorical variables are summarized as counts and percentages. Given the small sample size and descriptive nature of the study, no inferential statistical analyses were performed.

## 6 Ethics statement

This study was approved by the Ethics Committee of Children's Hospital of Soochow University. Written informed consent was waived due to the retrospective nature of the study and the use of anonymized data.

## 7 Results

### 7.1 Patient characteristics

During the study period (January 2018 to December 2025), a total of 1,138 pediatric patients with acute lymphoblastic leukemia (ALL) received chemotherapy at our institution, among whom six cases of radiologically confirmed cerebrovascular thrombosis were identified, corresponding to an incidence proportion of 0.53%. The median age at the time of cerebrovascular event was 10 years (range, 5–15 years).

**Figure 1:**
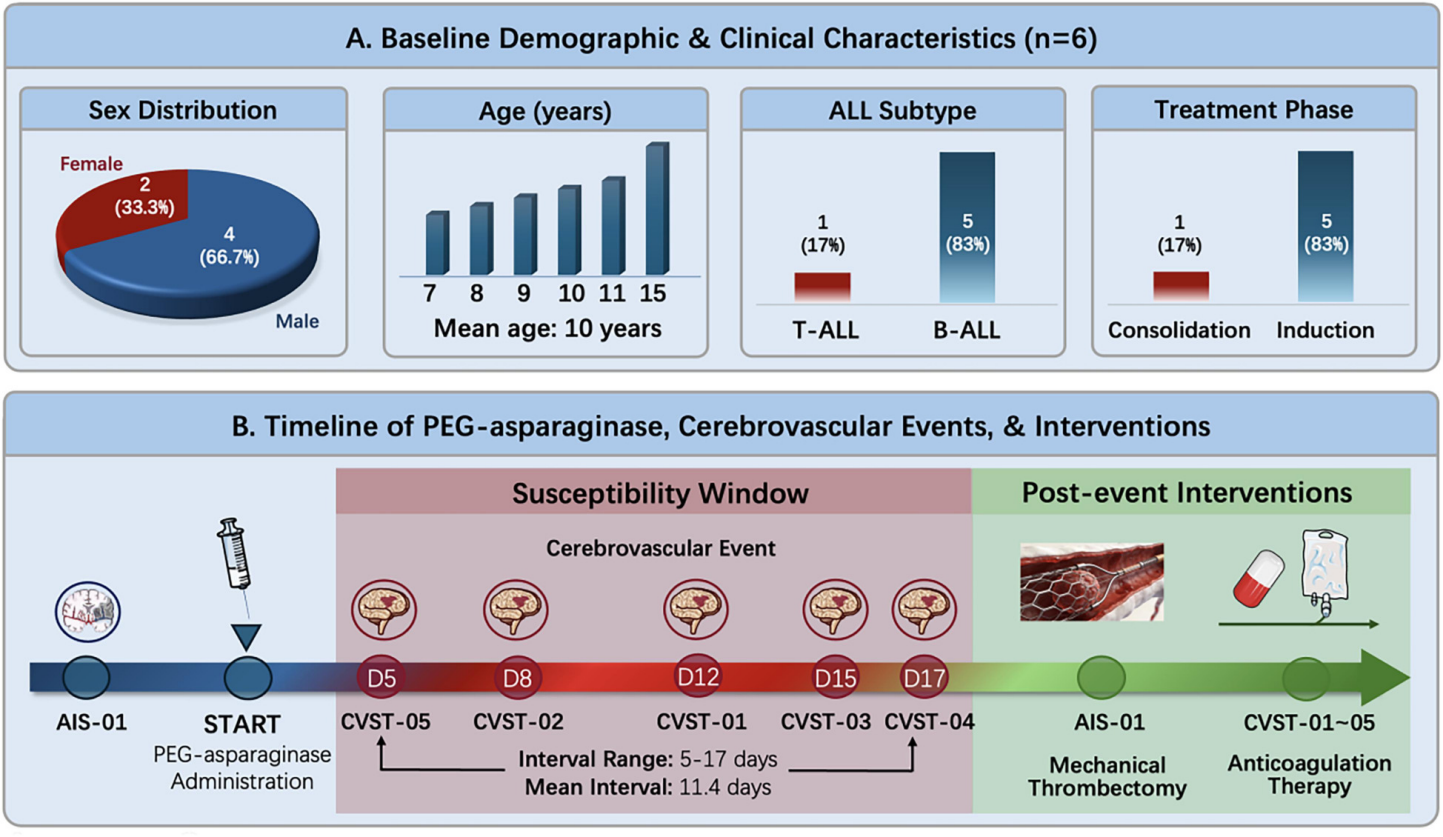
Timeline of PEG-asparaginase exposure, onset of cerebrovascular thrombosis, and coagulation recovery in pediatric ALL. **(A)** Distribution of cerebrovascular thrombosis events according to chemotherapy phase and PEG-asparaginase exposure. **(B)** Temporal relationship between PEG-asparaginase administration, symptom onset, anticoagulation therapy, and laboratory recovery.

Five of six events clustered 5–17 days after PEG-asparaginase exposure, whereas one AIS occurred during induction prior to PEG administration. Among the five PEG-asparaginase–exposed patients, the interval between the last dose and neurological symptom onset ranged from 5 to 17 days. Baseline demographic and clinical characteristics are summarized in Table 1. The temporal relationship between PEG-asparaginase administration, onset of cerebrovascular thrombosis events, and initiation of therapeutic interventions—highlighting the 5–17-day clustering window—is illustrated in Figure 1.

**Table 1:** Baseline demographic characteristics and chemotherapy exposure.

Case ID	Sex	Age (years)	ALL subtype	Treatment phase	PEG-asparaginase exposure	Interval from last PEG to event (days)	Corticosteroids	Central venous catheter
AIS-1	F	15	T-ALL	Induction	No	NA	Yes	No
CVST-1	F	9	B-ALL	Induction	Yes	12	Yes	Yes
CVST-2	M	11	B-ALL	Consolidation	Yes	8	Yes	No
CVST-3	M	10	B-ALL	Induction	Yes	15	Yes	No
CVST-4	M	7	B-ALL	Induction	Yes	17	Yes	No
CVST-5	M	8	B-ALL	Induction	Yes	5	Yes	No

ALL, acute lymphoblastic leukemia; B-ALL, B-cell acute lymphoblastic leukemia; T-ALL, T-cell acute lymphoblastic leukemia; AIS, arterial ischemic stroke; CVST, cerebral venous sinus thrombosis; PEG-asparaginase, polyethylene glycol–conjugated asparaginase; NA, not applicable.

### 7.2 Clinical presentation and neuroimaging findings

#### 7.2.1 Cerebral venous sinus thrombosis

Five patients presented with acute neurological symptoms, most commonly seizures and headache, and were diagnosed with cerebral venous sinus thrombosis (CVST). Brain MRI combined with magnetic resonance venography demonstrated thrombosis involving major dural venous sinuses in all cases.

#### 7.2.2 Acute arterial ischemic stroke

One patient presented with sudden-onset focal neurological deficits and was diagnosed with acute arterial ischemic stroke. Diffusion-weighted imaging revealed hyperintense lesions involving the left basal ganglia, temporal lobe, and parietal lobe, with corresponding hypointensity on apparent diffusion coefficient maps, consistent with acute infarction. Magnetic resonance angiography demonstrated occlusion of the left middle cerebral artery (M1 segment), while the contralateral arterial circulation remained patent.

**Figure 2:**
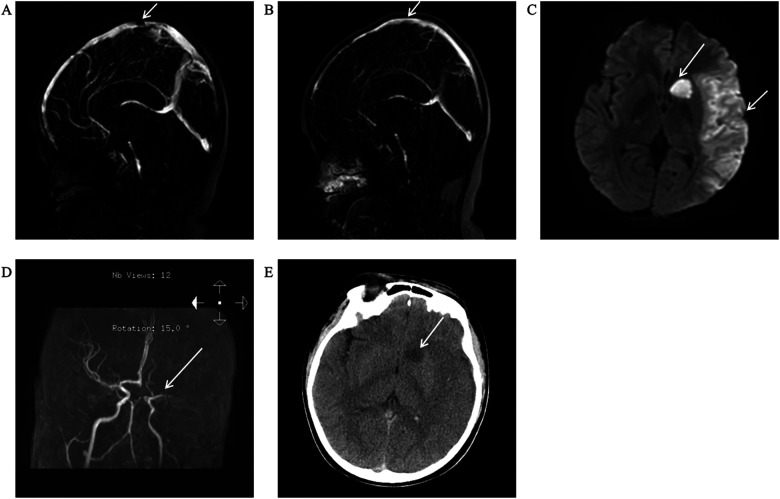
Representative neuroimaging findings of cerebrovascular thrombosis in pediatric acute lymphoblastic leukemia. **(A)** Magnetic resonance venography (MRV) demonstrating superior sagittal sinus thrombosis at symptom onset in patient CVST-5. **(B)** Follow-up MRV showing partial recanalization on subsequent imaging. **(C)** Diffusion-weighted imaging (DWI) revealing acute infarction in the left middle cerebral artery territory in patient AIS-1. **(D)** Magnetic resonance angiography (MRA) demonstrating occlusion of the left middle cerebral artery (M1 segment). **(E)** Post-thrombectomy computed tomography (CT) confirming vascular recanalization without hemorrhagic transformation.

A follow-up MRI performed one month later showed evolution of ischemic lesions without evidence of new infarction. Clinical manifestations and neuroimaging findings are summarized in Table 2. Representative neuroimaging findings of CVST and acute arterial ischemic stroke, including pre- and post-thrombectomy images, are shown in Figure 2.

**Table 2:** Clinical presentation and neuroimaging characteristics.

Case ID	Event date	Initial symptoms	Seizure	Altered consciousness	Event type	Vascular territory	Imaging modality
AIS-1	2024/6/15	Right-sided hemiplegia, facial palsy, impaired consciousness	No	Yes	AIS	Left MCA (M1)	MRI + MRA
CVST-1	2025/10/3	Seizure	Yes	No	CVST	SSS, transverse sinus	MRI + MRV
CVST-2	2025/3/11	Headache	No	No	CVST	SSS, sigmoid sinus	MRI + MRV
CVST-3	2023/7/27	Seizure, unsteady gait	Yes	Yes	CVST	Transverse sinus	MRI + MRV
CVST-4	2021/9/23	Seizure, right upper limb monoparesis	Yes	Yes	CVST	SSS	MRI + MRV
CVST-5	2023/8/24	Seizure	Yes	Yes	CVST	Transverse sinus	MRI + MRV

AIS, arterial ischemic stroke; CVST, cerebral venous sinus thrombosis; MCA, middle cerebral artery; M1, first segment of the middle cerebral artery; SSS, superior sagittal sinus; MRI, magnetic resonance imaging; MRA, magnetic resonance angiography; MRV, magnetic resonance venography.

At symptom onset, the platelet count of the AIS patient was 9 × 10⁹/L. Intravenous thrombolysis was not performed because pediatric AIS lacks standardized thrombolytic recommendations and the patient presented with severe thrombocytopenia. Emergency multidisciplinary consultation involving pediatric hematology, neurology, neurointervention, and intensive care specialists concluded that the risk of irreversible large-vessel infarction outweighed the hemorrhagic risk after platelet correction.

Platelet transfusion was administered prior to thrombectomy, resulting in a pre-procedural platelet count of 45 × 10⁹/L. Pre-procedural neuroimaging showed no evidence of intracranial hemorrhage or extensive established infarction. Mechanical thrombectomy was performed approximately 11.5 h after symptom onset and achieved successful vascular recanalization without hemorrhagic transformation on post-procedural imaging.

Therapeutic LMWH was initiated after repeat CT excluded intracranial hemorrhage and platelet counts recovered above 50 × 10⁹/L. During anticoagulation therapy, platelet counts were maintained between 25 and 81 × 10⁹/L with close hematologic monitoring. Follow-up imaging demonstrated stable infarct evolution without recurrent vascular occlusion.

## 8 Laboratory findings

At presentation, all patients demonstrated significantly elevated plasma D-dimer levels, ranging from 9,290 to 59,800 μg/L (institutional reference range <500 μg/L). Antithrombin III (AT-III) activity was reduced in all patients, with values ranging from 41.2% to 70.9% (reference range 80%–120%).

Platelet counts varied among patients, reflecting differences in chemotherapy phase and hematologic status at presentation. Prothrombin time and activated partial thromboplastin time showed no consistent abnormalities across cases. No laboratory or cerebrospinal fluid findings suggested central nervous system infection.

Among patients who received anticoagulation therapy, follow-up testing demonstrated recovery of AT-III activity in all treated cases, with initial increases observed within 5–13 days after treatment initiation. In several patients, AT-III activity returned to the normal range during the PEG-asparaginase-free interval. D-dimer levels declined markedly after initiation of therapy, with reductions observed within 1–3 days in most cases.

No patient received antithrombin III replacement therapy. Minor hemorrhagic transformation was observed in one patient who did not receive anticoagulation, without clinical deterioration or need for neurosurgical intervention. Detailed laboratory trends are summarized in Table 3**.** Follow-up coagulation testing was generally performed every 1–3 days during the acute hospitalization period according to clinical status and treatment response. Dynamic laboratory monitoring was performed more frequently during acute neurological deterioration or after initiation of anticoagulation therapy.

**Table 3:** Baseline coagulation parameters at symptom onset.

Case ID	D-dimer (µg/L)	AT-III activity (%)	Platelet count ( × 10⁹/L)	PT/APTT abnormal
AIS-1	22,080	55.9	9	No
CVST-1	59,800	50.1	109	No
CVST-2	9,950	50.6	421	No
CVST-3	21,990	41.2	294	No
CVST-4	9,290	50.7	159	No
CVST-5	15,880	70.9	140	No

AT-III, antithrombin III; PT, prothrombin time; APTT, activated partial thromboplastin time.

## 9 Treatment and outcomes

Therapeutic anticoagulation with low-molecular-weight heparin (LMWH) was administered to four patients with CVST. One CVST patient did not receive anticoagulation because of family refusal. Antiseizure medications were prescribed when clinically indicated.

The patient with acute arterial ischemic stroke underwent mechanical thrombectomy for large-vessel occlusion, resulting in successful recanalization and meaningful neurological improvement.

At the most recent follow-up, all six patients were alive. Most patients achieved favorable neurological outcomes, with no severe persistent neurological deficits. No recurrent cerebrovascular thrombosis events were observed during the available follow-up period. The median follow-up duration was 27 months (range, 8–48 months). Treatment strategies and clinical outcomes are summarized in Table 4.

**Table 4:** Treatment strategies, dynamic coagulation changes, and clinical outcomes.

Case ID	LMWH therapy	LMWH duration	Thrombectomy	AT-III recovery after treatment	Time to initial AT-III recovery (days)	AT-III normalization	D-dimer decline	Time to D-dimer decline (days)	PEG-asparaginase paused	PEG reintroduced	Hemorrhagic complication	Imaging outcome	Follow-up (months)	Neurological outcome	Recurrent thrombosis
AIS-1	Yes	14 weeks	Yes (MCA)	Yes	5	No	Yes	3	Yes	No	No	Recanalization	24	Residual hemiparesis	No
CVST-1	Yes	16 weeks	No	Yes	12	Yes	Yes	2	Yes	Yes	No	Recanalization	8	Full recovery	No
CVST-2	Yes	16 weeks	No	Yes	11	Yes	Yes	2	Yes	Yes	No	Recanalization	12	Full recovery	No
CVST-3	Yes	16 weeks	No	Yes	11	No	Yes	2	Yes	Switched asparaginase	No	Recanalization	36	Full recovery	No
CVST-4	No (refused)	NA	No	Yes (spontaneous)	7	Yes	Yes	3	Yes	Yes	Minor hemorrhagic transformation	No recanalization	48	Full recovery	No
CVST-5	Yes	12 weeks	No	Yes	12	No	Yes	1	Yes	Yes	No	Recanalization	30	Full recovery	No

AT-III recovery was defined as sustained post-treatment increase from nadir levels; normalization indicated return to the institutional reference range (80%–120%). Therapeutic LMWH was initiated after exclusion of intracranial hemorrhage and platelet recovery to clinically acceptable levels.

LMWH, low-molecular-weight heparin; AT-III, antithrombin III; PEG, PEG-asparaginase; MCA, middle cerebral artery; NA, not applicable.

## 10 Discussion

This single-center case series describes six pediatric patients with acute lymphoblastic leukemia (ALL) who developed cerebrovascular thrombosis during chemotherapy, including five cases of CVST and one AIS. Although one AIS event occurred prior to PEG-asparaginase exposure, five of six thrombotic events clustered within 5–17 days after PEG administration, suggesting a potentially actionable temporal clustering pattern. At symptom onset, all patients demonstrated elevated D-dimer levels and reduced antithrombin III activity. The marked D-dimer elevation likely reflects extensive activation of coagulation with secondary fibrinolysis, which is consistent with acute sinus or large-vessel thrombosis (15, 16). These findings suggest a disturbance of the hemostatic balance during intensive chemotherapy, which may contribute to thrombus formation. To our knowledge, few previous pediatric ALL series have simultaneously characterized temporal clustering after PEG-asparaginase exposure, dynamic AT-III recovery, and real-world thrombectomy management within a single cohort. The principal strengths of our study include the integration of temporal PEG-asparaginase exposure patterns, serial coagulation kinetics, and multidisciplinary real-world management data within a single pediatric ALL cohort.

Cerebrovascular thrombosis represents an uncommon but increasingly recognized complication of pediatric ALL therapy, with reported incidences ranging from 1% to 3%, most frequently during induction or early intensification phases (4, 5, 8, 17). Multicenter data have characterized common presentations, including headache, seizures, and focal deficits, yet mechanistic and management data remain limited (4, 8, 15). The incidence observed in our cohort was slightly lower than that reported in previous multicenter studies, which may relate to the small sample size, retrospective design, or underrecognition of subclinical thrombotic events. Our cohort adds detailed laboratory and imaging correlation, reinforcing the concept of temporal clustering following PEG-asparaginase exposure (11, 12, 15).

Asparaginase is known to impair hepatic protein synthesis, resulting in depletion of natural anticoagulants—particularly antithrombin III—and reduced fibrinogen levels (11). This induces a procoagulant shift that predisposes patients to thrombosis. Previous studies have shown that thrombotic events and antithrombin depletion commonly peak within one to two weeks after PEG-asparaginase exposure, likely reflecting delayed suppression of hepatic anticoagulant protein synthesis. The laboratory profile observed in our patients closely aligns with these reports, thereby supporting a biologically plausible treatment-related mechanism. Concurrent corticosteroid therapy has been demonstrated to further amplify thrombotic risk, particularly in intermediate- and high-risk protocols (8, 17). This may explain the observed temporal clustering of events during the initial treatment phases.

Beyond asparaginase effects, thrombogenesis in pediatric ALL is multifactorial. Chemotherapy-related endothelial injury, leukemia-associated systemic inflammation, central venous catheter placement, older age, high-risk disease phenotype, and intensive treatment regimens have all been implicated as risk modifiers (18). However, no single biomarker reliably predicts cerebrovascular events (8, 19).

Management of cerebrovascular events in pediatric ALL remains complex because anticoagulation must be balanced against thrombocytopenia, bleeding risk, and ongoing cytotoxic therapy (20). In our series, therapeutic low-molecular-weight heparin (LMWH) was generally well tolerated in patients with CVST, without major hemorrhagic complications despite chemotherapy-associated cytopenia (21). Patients who received anticoagulation demonstrated radiological recanalization and favorable neurological recovery, whereas incomplete venous recanalization was observed in the patient who did not receive anticoagulation. Although limited by sample size, this pattern aligns with prior pediatric reports suggesting that early anticoagulation may facilitate venous recanalization and reduce long-term sequelae. Notably, no recurrent thrombotic events were observed in patients who underwent PEG-asparaginase re-exposure during follow-up.

The occurrence of AIS during pediatric ALL therapy is uncommon, and the extant interventional data remain limited. In our cohort, one adolescent patient with large-vessel occlusion underwent emergency mechanical thrombectomy despite severe thrombocytopenia at presentation. Because untreated large-vessel occlusion carried a high risk of irreversible neurological injury, urgent multidisciplinary assessment was performed, and platelet transfusion support was administered prior to intervention. Mechanical thrombectomy was favored over intravenous thrombolysis because large-vessel occlusion was radiographically confirmed, whereas profound thrombocytopenia increased the theoretical hemorrhagic risk of systemic thrombolysis. Importantly, pre-procedural neuroimaging demonstrated no evidence of intracranial hemorrhage despite severe thrombocytopenia. No hemorrhagic transformation occurred following thrombectomy, and partial neurological recovery was achieved with residual hemiparesis at follow-up.

Emerging pediatric stroke literature suggests that thrombectomy may be considered in carefully selected children with large-vessel occlusion when performed within an appropriate therapeutic window by experienced multidisciplinary teams (22, 23). To our knowledge, reports describing mechanical thrombectomy during active pediatric ALL chemotherapy in the setting of severe thrombocytopenia remain limited. In our patient, successful recanalization was achieved without hemorrhagic transformation despite profound chemotherapy-associated thrombocytopenia, suggesting that endovascular intervention may be feasible in selected pediatric oncology patients after multidisciplinary risk assessment and hematologic optimization. Careful patient selection remains essential, particularly in the setting of chemotherapy-associated thrombocytopenia. In our patient, the absence of intracranial hemorrhage and extensive established infarction on pre-procedural imaging supported the decision to proceed with thrombectomy after platelet correction.

Dynamic coagulation recovery patterns following cerebrovascular thrombosis during pediatric ALL therapy have been insufficiently characterized in previous studies, particularly regarding serial AT-III and D-dimer kinetics after treatment initiation. In our cohort, serial monitoring demonstrated gradual recovery of AT-III activity within 5–13 days after anticoagulation, whereas D-dimer levels declined rapidly within 1–3 days following treatment initiation or thrombectomy. In several patients, normalization of AT-III activity occurred during the PEG-asparaginase–free interval, supporting the reversibility of treatment-related coagulopathy. Notably, low-molecular-weight heparin alone was sufficient to facilitate coagulation recovery in all treated patients, and none required AT-III replacement therapy. These observations suggest that serial assessment of AT-III activity and D-dimer may provide not only diagnostic information but also dynamic biomarkers of therapeutic response and coagulation recovery (15).

Based on the reproducible temporal clustering observed in our cohort, a pragmatic surveillance approach may be clinically reasonable during the 5–17-day period following PEG-asparaginase exposure. In patients presenting with new neurological symptoms—particularly headache or seizure—targeted monitoring of coagulation parameters during this interval may facilitate earlier recognition of cerebrovascular thrombosis. Serial assessment of D-dimer and antithrombin III activity, combined with prompt neuroimaging evaluation for altered consciousness or focal neurological deficits, may support timely diagnosis and intervention.

This study has several limitations. Its retrospective design and small sample size preclude causal inference and robust risk modeling. Comprehensive thrombophilia screening and longitudinal coagulation profiling were not uniformly available. Nevertheless, the integration of laboratory abnormalities, detailed neuroimaging, and clinical outcomes provides clinically meaningful insight into this infrequent but serious complication. The timing of serial coagulation measurements was not standardized, which may have influenced the observed kinetics of AT-III recovery and D-dimer decline.

In summary, cerebrovascular thrombosis during pediatric ALL therapy in our cohort frequently occurred within 5–17 days after PEG-asparaginase exposure and was associated with reproducible reductions in antithrombin III activity and marked D-dimer elevation. These findings support heightened clinical vigilance during this high-risk interval, particularly in children presenting with new neurological symptoms. Dynamic coagulation monitoring combined with timely neuroimaging evaluation may facilitate earlier diagnosis and therapeutic intervention. These observations may help inform future surveillance strategies during the early post–PEG-asparaginase period. Larger prospective multicenter studies are warranted to validate these observations and to determine whether targeted surveillance strategies may improve risk stratification and clinical outcomes.

## Data Availability

The raw data supporting the conclusions of this article will be made available by the authors, without undue reservation.
